# 587. Impact of the infectious site infection with Staphylococcus aureus in Europe: results from the economics of the SALT study

**DOI:** 10.1093/ofid/ofae631.182

**Published:** 2025-01-29

**Authors:** Jon Salmanton-Garcia, Caroline Bruns, Jule Rutz, Markus Albertsmeier, Juliane Ankert, Louis Bernard, Camille Bataille, Elodie Couvé-Deacon, María Fernández-Ferrer, Jesús Fortún, Alicia Galar, Eva Grill, Thomas Guimard, Jörg-Janne Vehreschild, Jannik Stemler, Jan-Hendrik Naendrup, Jürgen Hampl, Bradly Tallon, Rosanne Sprute, Juan Pablo Horcajada, Joan Mollar-Maseres, Patricia Muñoz, Mathias Pletz, Ferdinand Serracino-Inglott, Alex Soriano, Tim Vilz, Harald Seifert, Oliver A Cornely, Sibylle Mellinghoff, Blasius Liss, Sebastian Wingen-Heimann

**Affiliations:** University Hospital Cologne, Cologne, Germany, Cologne, Nordrhein-Westfalen, Germany; University Hospital Cologne, Cologne, Nordrhein-Westfalen, Germany; University Hospital Cologne, Cologne, Nordrhein-Westfalen, Germany; Ludwig-Maximilians-Universität Munich, Munich, Bayern, Germany; University Hospital Jena, Jena, Thuringen, Germany; CHU TOURS, TOURS, Centre, France; Université Limoges, Limoges, Limousin, France; Université Limoges, Limoges, Limousin, France; Universitat Pompeu Fabra, Bacelona, Catalonia, Spain; Ramón y Cajal University Hospital, Madrid, Madrid, Spain; Hospital General Universitario Gregorio Marañón. Clinical Microbiology and Infectious Diseases Department. Instituto de Investigación Sanitaria Gregorio Marañón, Madrid, Madrid, Spain; Ludwig-Maximilians-Universität Munich, Munich, Bayern, Germany; CHD VENDEE, LA ROCHE / YON, Pays de la Loire, France; University of Cologne, Cologne, Nordrhein-Westfalen, Germany; University Hospital Cologne, Cologne, Nordrhein-Westfalen, Germany; University of Cologne, Cologne, Nordrhein-Westfalen, Germany; University of Cologne, Cologne, Nordrhein-Westfalen, Germany; Manchester University NHS Foundation Trust, Manchester, England, United Kingdom; University Hospital Cologne, Cologne, Nordrhein-Westfalen, Germany; Hospital del Mar, Barcelona, Catalonia, Spain; La Fe University and Polytechnic Hospital, Valencia, Comunidad Valenciana, Spain; Hospital General Universitario Gregorio Marañón, Madrid, Madrid, Spain; University Hospital Jena, Jena, Thuringen, Germany; Manchester University NHS Foundation Trust, Manchester, England, United Kingdom; Hospital Clínic de Barcelona, Barcelona, Catalonia, Spain; University Hospital Bonn, Bonn, Nordrhein-Westfalen, Germany; Institute for Medical Microbiology, Immunology and Hygiene, University of Cologne, Cologne, Nordrhein-Westfalen, Germany; University of Cologne, Faculty of Medicine and University Hospital Cologne, Cologne, Nordrhein-Westfalen, Germany; University Hospital of Cologne, Cologne, Nordrhein-Westfalen, Germany; Helios University Wuppertal, Wuppertal, Nordrhein-Westfalen, Germany; University Hospital Cologne, Cologne, Nordrhein-Westfalen, Germany

## Abstract

**Background:**

Surgical site infections (SSI), mainly caused by Staphylococcus aureus, represent a significant economic burden in Europe, leading to an increase in length of hospitalization, mortality and treatment costs, especially with drug-resistant strains. such as methicillin-resistant Staphylococcus aureus. A new case-control study in high-volume centers aims to provide detailed data on the economic impact of treating Staphylococcus aureus SSIs.

**Methods:**

The SALT study is a multinational retrospective cohort study with a case-control analysis focused on Staphylococcus aureus SSIs in Europe. The study included participants from France, Germany, Italy, Spain, and the United Kingdom who underwent invasive surgery in 2016 and employed a microcosting approach to assess health economic factors, matching Staphylococcus aureus SSI cases with controls.

**Results:**

In 2016, among 178,904 surgical patients in five European countries, 764 developed Staphylococcus aureus SSI. By matching 744 cases with controls, the study revealed that Staphylococcus aureus SSI cases incurred higher immediate hospitalization costs (€8,809.9), compared to controls (€6,032.4). Furthermore, Staphylococcus aureus SSI cases exhibited higher costs for readmissions within the first year after surgery (€7,961.6 versus €5,298.6), with significant differences observed. Factors associated with an increase in surgery-related costs included the cost of hospitalization immediately after surgery, first ICU admission within 12 months, and hospital readmission within 12 months, as identified by analysis multivariable.

**Conclusion:**

The higher rates of hospitalization, ICU admissions, and readmissions among Staphylococcus aureus SSI cases highlight the severity of these infections and their impact on healthcare costs, emphasizing the potential benefits of infection control measures based on evidence and improved patient care to mitigate the economic burden.

**Disclosures:**

**Thomas Guimard, MD**, Gilead: Convention invitation|Menarini France: Hospitality|MSD: Hospitality|Pfizer: Advisor/Consultant|Shionogi BV: Hospitality **Oliver A. Cornely, Prof. Dr.**, Abbott: Honoraria|Abbvie: Advisor/Consultant|Abbvie: Honoraria|AiCuris: Advisor/Consultant|Akademie fur Infektionmedizin: Honoraria|Al-Jazeera Pharmaceuticals/Hikma: Honoraria|amedes: Honoraria|AstraZeneca: Honoraria|Basilea: Advisor/Consultant|Biocon: Advisor/Consultant|BMBF: Grant/Research Support|Boston Strategic Partners: Advisor/Consultant|CIdara: Advisor/Consultant|CIdara: Expert Testimony|CIdara: Grant/Research Support|CIdara: Participation on a DRC or DSMB|CoRe Consulting: Stocks/Bonds (Private Company)|Deutscher Arzteverlag: Honoraria|DZIF: Grant/Research Support|EasyRadiology: Stocks/Bonds (Private Company)|EU-DG RTD: Grant/Research Support|F2G: Grant/Research Support|Gilead: Advisor/Consultant|Gilead: Grant/Research Support|Gilead: Honoraria|Grupo Biotoscana/United Medical/Knight: Honoraria|GSK: Advisor/Consultant|GSK: Honoraria|IQVIA: Advisor/Consultant|IQVIA: Participation on a DRC or DSMB|Janssen: Advisor/Consultant|Janssen: Participation on a DRC or DSMB|Matinas: Advisor/Consultant|MedPace: Advisor/Consultant|MedPace: Grant/Research Support|MedPace: Participation on a DRC or DSMB|Medscape/WebMD: Honoraria|MedUpdate: Honoraria|Menarini: Advisor/Consultant|Moderna: Honoraria|Molecular Partners: Advisor/Consultant|MSD: Grant/Research Support|MSD: Honoraria|MSG-ERC: Advisor/Consultant|Mundipharma: Advisor/Consultant|Mundipharma: Grant/Research Support|Mundipharma: Honoraria|Noscendo: Honoraria|Noxxon: Advisor/Consultant|Octapharma: Advisor/Consultant|Octapharma: Grant/Research Support|Pardes: Advisor/Consultant|Partner Therapeutics: Advisor/Consultant|Patent: US18/562644|Paul-Martini-Stiftung: Honoraria|Pfizer: Advisor/Consultant|Pfizer: Grant/Research Support|Pfizer: Honoraria|PSI: Advisor/Consultant|PSI: Participation on a DRC or DSMB|Pulmocide: Participation on a DRC or DSMB|Sandoz: Honoraria|Scynexis: Advisor/Consultant|Scynexis: Grant/Research Support|Seqirus: Advisor/Consultant|Seqirus: Honoraria|Seres: Advisor/Consultant|Shionogi: Advisor/Consultant|Shionogi: Honoraria|streamedup!: Honoraria|The Prime Meridian Group: Advisor/Consultant|Touch Independent: Honoraria|Vitis: Honoraria

